# Supplementary material for: Barriers to getting into postgraduate specialty training for junior Australian doctors: An interview-based study
Source: PLoS One. 2021 Oct 21;16(10):e0258584. doi: 10.1371/journal.pone.0258584 (PMC8530333; doi:10.1371/journal.pone.0258584)
Supplement: S1 File — (DOCX) [file pone.0258584.s001.docx]

**Supplementary information 1:** Interview guide

| **Question** | **Specific prompts** | **General prompts** |
| --- | --- | --- |
| Could we start by you telling me a little about yourself and your career as a doctor? | Things like your Current practice location, Area of Medicine, Stage of medical career e.g. pre-registrar, registrar, and where you did each stage of your medical training? | Could you please expand on that?  That is very interesting, could you tell me more?  Really, what was that like?  Reflecting on that time in X, could you give me a bit more detail about X experience? |
| What are the major factors that have influenced your medical career journey to date? | Identify factors that influenced participant’s career decision; getting into a chosen specialty; current practice location; decision-making in the context of family situations, partner employment, incentives, professional support |  |
| What were the important time points when things happened that determined the current shape of your medical career, including getting into specialty training? |  |  |
| What made these time points important? |  |  |
| What happened at those times and how did they affect you? |  |  |
| Have you had to move from where you were living to pursue a training opportunity, or to meet clinical/professional college requirements? |  |  |
| Did you later return to where you were? |  |  |
| Have you had breaks in practice? |  |  |
| Can you tell me the reasons for those breaks? |  |  |
| What would have made your medical career progression better informed? |  |  |
| What (else) would have improved the way your medical career has progressed? |  |  |
