# Supplementary material for: Barriers to getting into postgraduate specialty training for junior Australian doctors: An interview-based study
Source: PLoS One. 2021 Oct 21;16(10):e0258584. doi: 10.1371/journal.pone.0258584 (PMC8530333; doi:10.1371/journal.pone.0258584)
Supplement: Attachment — Submitted filename: PLOS_peerreview_120521.docx [file pone.0258584.s003.docx]

Many thanks for the opportunity to review this manuscript, the methodology is sound and the conclusions in the most part seem reasonable. However, I think they could be clearer at times. I also am not sure about their conclusion regarding GP training. In addition I wonder if more explicitly linking their findings to the theory that underpins this may help make the paper clearer, and recommendations easier to implement.

Major points

1. I recognise the aim of this research - exploring an at times opaque process that can cause doctors significant stress and may not result in the most efficient use of resources. To me one of the fundamental reasons for this is that there may be less need for specialists than people who want to be them. I feel another recommendation therefore is the need for clear guidelines and feedback for young doctors, a recognition in their training they may not get to do their first choice, and support for them to change their goals. This is touched on but I wonder if the need for structures and support to particularly help doctors that may not be able to persue their original first choice career is important to be a stand alone recommendation in it’s own right?
2. Speciality training, and promotion in medicine in general, has in the past very much relied on who you know, and hidden "old boys" networks. The data seems to suggest (though I could be wrong) there is an element of that still happening; there are implicit barriers regarding who to make yourself known to, and exactly what is needed on CVs for the next stage. I think this potentially is a very important finding as it means the selection process is still likely to be highly influenced by bias. I bring this up because I suspect some of the specialist's argument may be that the barriers in place make sure that only the best persevere, so are valuable. From reading your paper your data suggests it could be argued instead that talented people are struggling because the pathways aren't clear resulting in ongoing implicit barriers (regarding knowing the right people, getting the right information on hearsay). I know the study didn’t set up to do this but was there any evidence that this may disadvantage some groups more than others? If not it may still be worth bringing this out a bit more and being clear that this ongoing bias because of complex and changing systems is unlikely to be good for the system, as well as causing unnecessary stress.
3. As a GP I struggle with your wording regarding the GP speciality training. I recognise this could just be my personal interpretation but it feels slightly patronising to say that a way to increase GP numbers is to tell people its not difficult to get in. This idea that general practice is an “easy” option is one that GPs have been struggling with for years now. It is just my opinion, but that wording doesn’t feel helpful. Your paper focuses on barriers so maybe your didn’t get a chance to explore this but I wonder if actually your results suggest a “success story” for general practice. Is it just that the bar is set low? I don’t know the Australia situation but in the UK the college has developed clear national criteria, transparent vetting and an in-person selection process that is transparent, with multiple pre-defined stations (consultation, group discussion, reflective exercise) that is clearly set out for people before they attend. My experience would fit with what you are saying, that my training flowed from one stage to the next, but that was in part because it was clear what was needed at each one. Could the lack of stress in the GP trainees actually be because your Australian college has developed a clear pipeline that is easy for GPs to follow. I accept that the larger number of training places makes a difference but is it just that? Are there things the specialities could learn from the way the generalists recruit for training?
4. You mentioned that you were using action theory to understand your results, it may be helpful for that to be a bit more explicit. How do your themes and recommendation relate to each of the four aspects? Is there one of them that is a particular problem, or are the barriers in each section equal.

Minor

Line 64 – is there evidence that speciality training places are being limited despite them needing more people? My understanding is that places are limited by the need for them based on population requirements (though this could be a UK experience). Globally there is a real issue of over-specialisation and I would assume that the issue with difficulty getting into some specialities is because there are more people who want to be certain specialists than we as the population need? Which in turn means there may be a need for people to create support to help people look at other options is they don’t get into their speciality of choice.

Line 84-86: I disagree with what you think the choice to take a break after foundation training in the UK suggests. I know many people who did that and almost all of them did so either because they wanted to take the opportunity to travel when they were young and didn’t have family commitments, or they didn’t know exactly what they wanted to specialise in so they wanted to take the time to do some more general jobs before they committed to a speciality. Given there is scant research in this area I am not sure you can say this suggests that doctors are taking breaks because of stress/exhaustion or to enhance CVs.

Line 353-359: I think this is an interesting recommendation because it sounds very similar to the UK system: a national structured system for each speciality with clear criteria describing what is expected at each level, which every speciality has set out their career progression. It certainly isn’t perfect, and there are issues however, have you looked at other international options ? Your study is in the Australian context, and I recognise it is difficult to take qualitative research out of context – but it may be interesting to see how other healthcare systems compare with your recommendations?

I am not clear exactly how you targeted your population – how did you get your mix of interviewees, did you email/send a letter, how did you choose who to email. Also, how many people did you contact? How many refused to take part? Were there any particular characteristics in those who didn’t take part that differ from those you did interview?

I am not clear what you mean by a mix of locations – is this geographical or is related to town size/rurality (which I imagine is a big issue in Australia)?

I would appreciate a bit more information on the interviewees. You talk about a mix of locations, what do you mean by that. Also it may be valuable to know what specialities were represented.
