# Supplementary material for: Barriers to getting into postgraduate specialty training for junior Australian doctors: An interview-based study
Source: PLoS One. 2021 Oct 21;16(10):e0258584. doi: 10.1371/journal.pone.0258584 (PMC8530333; doi:10.1371/journal.pone.0258584)
Supplement: Attachment — Submitted filename: PLOS_Response to reviewer table_11June2021.docx [file pone.0258584.s004.docx]

Response to PLOS ONE Decision: Revision required [PONE-D-21-08376] - [EMID:c4d14748b95338e0]

|  | **Reviewer comment** | **Response** |
| --- | --- | --- |
| 1 | Many thanks for the opportunity to review this manuscript | Thank you for this comment |
| 2 | the methodology is sound and the conclusions in the most part seem reasonable. | Thank you for this positive feedback |
| 3 | I wonder if more explicitly linking the findings to the theory that underpins this may help make the paper clearer, and recommendations easier to implement. | Thank you for this suggestion, see our response at comment 9. |
| 4 | I recognise the aim of this research - exploring an at times opaque process that can cause doctors significant stress and may not result in the most efficient use of resources. | Thank you for this positive comment |
| 5 | To me one of the fundamental reasons for this is that there may be less need for specialists than people who want to be them. | Our research explores the doctors’ perspectives, rather than the system need but we raise more about this in the introduction and the discussion, based on your suggestion, thank you.  **Introduction**  To some extent, tight criteria and limited places in some specialty fields are necessary as there may be less need for sub-specialists and specialists than doctors who want to pursue non-GP specialty fields. Countering this, there are a greater number of places in general practice training, where there is known to be strong community need to bolster primary care service capacity, but the degree to which pre-registrar doctors still find getting into general practice difficult, is yet to be researched.  **Discussion**  The former group had a relatively seamless entry to specialty training and good career control, potentially as the most available places exist in GP training of any specialty field but also potentially because the training program entry is clearly structured to articulate with the generalist nature of a pre-registrar’s experience to date. This access to general practice careers is important for the community and could play a part in bolstering of primary care service capacity, particularly where it is combined with campaigns that showcase general practice as a sophisticated career option which provides for strong status in the community [17]. |
| 6 | I feel another recommendation … is the need for clear guidelines and feedback for young doctors, a recognition in their training they may not get to do their first choice, and support for them to change their goals. This is touched on but I wonder if the need for structures and support to particularly help doctors that may not be able to pursue their original first choice career is important to be a standalone recommendation in it’s own right? | Thank you for this suggestion, we agree with its sentiment.  In the **Discussion w**e have added:  Pre-registrars may need this guidance at every point in the process of pursuing specialty training goals, but particularly to help them re-evaluate specialty training options and supporting them to change their goals if they are struggling to gain entry to a particular college. This is essential where pre-registrars may lack a whole of system view and therefore may benefit from mature mentorship, independent of a specialty college. |
| 7 | Speciality training, and promotion in medicine in general, has in the past very much relied on who you know, and hidden "old boys" networks. The data seems to suggest (though I could be wrong) there is an element of that still happening; there are implicit barriers regarding who to make yourself known to, and exactly what is needed on CVs for the next stage. I think this potentially is a very important finding as it means the selection process is still likely to be highly influenced by bias. I bring this up because I suspect some of the specialist's argument may be that the barriers in place make sure that only the best persevere, so are valuable. From reading your paper your data suggests it could be argued instead that talented people are struggling because the pathways aren't clear resulting in ongoing implicit barriers (regarding knowing the right people, getting the right information on hearsay). I know the study didn’t set up to do this but was there any evidence that this may disadvantage some groups more than others? If not it may still be worth bringing this out a bit more and being clear that this ongoing bias because of complex and changing systems is unlikely to be good for the system, as well as causing unnecessary stress. | Thank you for this suggestion. We have expanded our Discussion to raise some of these important factors that are not always clearly recognised.  In the **Discussion** we added:  The implications are that these barriers may intersect over many years for pre-registrar doctors who are not successful at getting into specialty training, potentially diminishing their morale and resilience. This may require that hospitals and health services embrace training-related pressures as part of their well-being policies for junior doctors. They also require the training system to recognise that pathways are not always clear and may include real or perceived selection biases to selection based on who is known within the specialty network or not. There may also be a narrow view of applicable skills which mismatches the trainee’s experiences, each causing unnecessary stress. |
| 8 | As a GP I struggle with your wording regarding the GP speciality training. I recognise this could just be my personal interpretation but it feels slightly patronising to say that a way to increase GP numbers is to tell people its not difficult to get in. This idea that general practice is an “easy” option is one that GPs have been struggling with for years now. It is just my opinion, but that wording doesn’t feel helpful. Your paper focuses on barriers so maybe you didn’t get a chance to explore this but I wonder if actually your results suggest a “success story” for general practice. Is it just that the bar is set low? I don’t know the Australia situation but in the UK the college has developed clear national criteria, transparent vetting and an in-person selection process that is transparent, with multiple pre-defined stations (consultation, group discussion, reflective exercise) that is clearly set out for people before they attend. My experience would fit with what you are saying, that my training flowed from one stage to the next, but that was in part because it was clear what was needed at each one. Could the lack of stress in the GP trainees actually be because your Australian college has developed a clear pipeline that is easy for GPs to follow. I accept that the larger number of training places makes a difference but is it just that? Are there things the specialities could learn from the way the generalists recruit for training? | Thank you for noting this, we have tried to change this emphasis to ensure that apart from its seamless entry, this is not meant to be reflective of it being a ‘lesser’ choice.  **Discussion**  This access to general practice careers is important for the community and could play a part in bolstering of primary care service capacity, particularly where it is combined with campaigns that showcase general practice as a sophisticated career option with strong status in the community [17]. |
| 9 | You mentioned that you were using action theory to understand your results, it may be helpful for that to be a bit more explicit. How do your themes and recommendation relate to each of the four aspects? Is there one of them that is a particular problem or are the barriers in each section equal? | Thank you for this suggestion.  Firstly, we made it clearer in the Introduction, about the stages of action theory. This only required minor editing.  Secondly, we then more closely discussed the results in relation to these stages to propose various implications. Our sense is that this strengthened the article.  **Discussion**  For those pursuing non-GP specialties, they faced multiple challenges that resonated across various fields, including surgery and internal medicine. These are appraised through the lens of work action theory [14].  At the stage of goal development and orientation, pre-registrar doctors aiming for non-GP fields evaluated competition and sought to orientate themselves to selected specialty training systems relevant to themselves, but they faced a lack of clear information about the range of non-GP training options. Instead, they noted complex and changing information about requirements, mostly sourced through peers.  At the stage of plan development and execution, pre-registrar doctors undertook early and active planning and executed these plans purposefully and methodically to achieve entry to non-GP specialties. This entailed additional non-clinical workload and relocation requirements which most could absorb in the name of achieving the career goal. However, some doctors may find these requirements hard to meet depending on the social and economic cost they might impose, including disrupting their connection to a place and scope of clinical work as well as to family or community resources. This may be case for doctors engaged interested in staying attached to rural medicine, if they are required to relocate to metropolitan tertiary hospitals for a training-related goal.  At the stage of executing the plan and processing feedback…, |
| 10 | Line 64 – is there evidence that speciality training places are being limited despite them needing more people? My understanding is that places are limited by the need for them based on population requirements (though this could be a UK experience). Globally there is a real issue of over-specialisation and I would assume that the issue with difficulty getting into some specialities is because there are more people who want to be certain specialists than we as the population need? Which in turn means there may be a need for people to create support to help people look at other options is they don’t get into their speciality of choice**.** | Thank you for noting this.  We expanded this section in the Introduction.  **Introduction**  Such evidence would be useful for developing more tailored support for early-career doctors and informing the design of postgraduate training systems for specialty entry across the balance of specialties needed by the community.  …  To some extent, tight criteria and limited places in some specialty fields are necessary as there may be less need for sub-specialists and specialists than doctors who want to pursue non-GP specialty fields. Countering this, there are a greater number of places in general practice training, where there is known to be strong community need to bolster primary care service capacity, but the degree to which pre-registrar doctors find getting into general practice difficult, is yet to be researched.  **Discussion**  We already had the following text, which has been expanded (see comment 6)  Another avenue to improve system design includes increasing formal career guidance and professional supports for doctors throughout the first 2-5 years of practise [8]. Critically this advice should reduce the need to source information from peers a few years ahead, which is not necessarily accessible, sensitive, or objective in nature. Pre-registrars may need this guidance at every point in the process of pursuing specialty training goals, but particularly to help them re-evaluate specialty training options and supporting them to change their goals if they are struggling to gain entry to a particular college. This is essential where pre-registrars may lack a whole of system view and therefore may benefit from mature mentorship, independent of a specialty college. Finally, hospital and health services could reduce the need for pre-registrars to relocate if they engaged more local up-skilling and networking with specialist clinical groups for emerging doctors with clinical networks. |
| 11 | Line 84-86: I disagree with what you think the choice to take a break after foundation training in the UK suggests. I know many people who did that and almost all of them did so either because they wanted to take the opportunity to travel when they were young and didn’t have family commitments, or they didn’t know exactly what they wanted to specialise in so they wanted to take the time to do some more general jobs before they committed to a speciality. Given there is scant research in this area I am not sure you can say this suggests that doctors are taking breaks because of stress/exhaustion or to enhance CVs. | Thank you for your comment. We have added more detail at line 88 **Introduction** to show that stress and exhaustion were key findings of the referenced study that was done. We acknowledge your anecdotal evidence that this is also about travelling and career decisions. We note that these doctors were still working (rather than just travelling), they were just not moving into specialty training. Some of the reason for not moving straight to specialty training was also related to not having career control and needing more time to prepare for specialty entry.  One interview-based study of fourteen doctors suggested taking a break from training in foundational year 3, rather than entering specialty training, related to training-work challenges at this stage such as exhaustion, stress, needing more time to prepare competitive portfolios for entering specialty training, and because they sensed a lack of control at this career stage |
| 12 | Line 353-359: I think this is an interesting recommendation because it sounds very similar to the UK system: a national structured system for each speciality with clear criteria describing what is expected at each level, which every speciality has set out their career progression. It certainly isn’t perfect, and there are issues however, have you looked at other international options? Your study is in the Australian context, and I recognise it is difficult to take qualitative research out of context – but it may be interesting to see how other healthcare systems compare with your recommendations? | Thank you, we have clarified this point with relation to its global relevance…  **Discussion**  …This differs from the current situation in Australia, where each college hosts this information, with varying degrees of clarity and updating. Such systems may already be available in other countries, from which Australia could draw. |
| 13 | I am not clear exactly how you targeted your population – how did you get your mix of interviewees, did you email/send a letter, how did you choose who to email. Also, how many people did you contact? How many refused to take part? Were there any particular characteristics in those who didn’t take part that differ from those you did interview? | Thank you for this suggestion  **Methods**  We added  This was done using maximum variation sampling involving emailing a wide sample using their last known email address, whereby 82 responded from which we selected participants of varying characteristics for interview.  The **results** go on to say..  Overall, 32 participants were included in our study until saturation was reached: 50% were female; 63% targeted non-GP specialties and were spread across as current pre-registrars (25%), registrars (31%) or recently fellowed as qualified specialists (44%).  This fits with the objectives of the study to explore a wide range of perspectives. |
| 14 | I am not clear what you mean by a mix of locations – is this geographical or is related to town size/rurality (which I imagine is a big issue in Australia)?  I would appreciate a bit more information on the interviewees. You talk about a mix of locations, what do you mean by that. Also it may be valuable to know what specialities were represented. | **Results**  We added geographic location |
