# Supplementary material for: Barriers to getting into postgraduate specialty training for junior Australian doctors: An interview-based study
Source: PLoS One. 2021 Oct 21;16(10):e0258584. doi: 10.1371/journal.pone.0258584 (PMC8530333; doi:10.1371/journal.pone.0258584)
Supplement: Attachment — Submitted filename: Response to Reviewer PlosOne_8Jul2021.docx [file pone.0258584.s006.docx]

Dear Editor of PlosONE

**Reference:** PONE-D-21-08376.

Thank you for recently peer reviewing our article ‘*Barriers to getting into postgraduate specialty training for junior doctors: an interview-based study*’. Please note that we have updated the title to include the country of the study.

We have also addressed each of the editorial points, namely:

We have included the study period of the interview data collection at line 26 and line 126.

- Removed a comma at line 71.
- Fixed a grammatical error at line 88.
- Fixed the sentence about action theory at line 95.
- Removed the interview guide at line 136 and placed this in the supplementary file 1.
- Included author initials at line 161.
- We have responded to each of the reviewer’s comments which we believe has substantially strengthened the paper.
- Uploaded new supplementary file 1.

We thank you for considering our manuscript and we look forward to hearing from you.

Kind regards

Dr Belinda O’Sullivan (Corresponding Author)
