# Supplementary material for: Barriers to getting into postgraduate specialty training for junior Australian doctors: An interview-based study
Source: PLoS One. 2021 Oct 21;16(10):e0258584. doi: 10.1371/journal.pone.0258584 (PMC8530333; doi:10.1371/journal.pone.0258584)
Supplement: Attachment — Submitted filename: Response to editor_26Jul2021.docx [file pone.0258584.s009.docx]

Response to editor - PONE-D-21-08376R3

Line 24 – we added ‘this study explored’

Line 48 – we removed the :

Line 134, we agreed with your suggestion and added ‘schedule informed by a topic guide’ and added ‘File’ to S1, as you suggested

Line 177 - we added ‘S2 File’ as you suggested

Line 405 – we added Supporting information as you suggested

We have checked the funding information but we could not find the financial disclosure information. We have corrected the funding information so that this is correct. This work was unfunded, but we added to the acknowledgements ‘*We acknowledge the support of the Australian government Department of Health’s Rural Health Multidisciplinary Training Program (RHMT program) which provides funding for Rural Clinical Schools and employed the researchers who did this work.’*
